# Correlations between Income Inequality and Antimicrobial Resistance

**DOI:** 10.1371/journal.pone.0073115

**Published:** 2013-08-29

**Authors:** Andrew Kirby, Annie Herbert

**Affiliations:** 1 Department of Microbiology, Leeds Teaching Hospitals National Health Service Trust, Leeds, West Yorkshire, United Kingdom; 2 Department of Research and Development, Pennine Acute National Health Service Trust, Manchester, Greater Manchester, United Kingdom; University of Ottawa, Canada

## Abstract

**Objectives:**

The aim of this study is to investigate if correlations exist between income inequality and antimicrobial resistance. This study’s hypothesis is that income inequality at the national level is positively correlated with antimicrobial resistance within developed countries.

**Data collection and analysis:**

Income inequality data were obtained from the Standardized World Income Inequality Database. Antimicrobial resistance data were obtained from the European antimicrobial Resistance Surveillance Network and outpatient antimicrobial consumption data, measured by Defined daily Doses per 1000 inhabitants per day, from the European Surveillance of antimicrobial Consumption group. Spearman’s correlation coefficient (r) defined strengths of correlations of: > 0.8 as strong, > 0.5 as moderate and > 0.2 as weak. Confidence intervals and *p* values were defined for all *r* values. Correlations were calculated for the time period 2003-10, for 15 European countries.

**Results:**

Income inequality and antimicrobial resistance correlations which were moderate or strong, with 95% confidence intervals > 0, included the following. *Enterococcus faecalis* resistance to aminopenicillins, vancomycin and high level gentamicin was moderately associated with income inequality (*r*= ≥0.54 for all three antimicrobials). *Escherichia coli* resistance to aminoglycosides, aminopenicillins, third generation cephalosporins and fluoroquinolones was moderately-strongly associated with income inequality (r= ≥0.7 for all four antimicrobials). *Klebsiella pneumoniae* resistance to third generation cephalosporins, aminoglycosides and fluoroquinolones was moderately associated with income inequality (r= ≥0.5 for all three antimicrobials). *Staphylococcus aureus* methicillin resistance and income inequality were strongly associated (r=0.87).

**Conclusion:**

As income inequality increases in European countries so do the rates of antimicrobial resistance for bacteria including *E. faecalis, E. coli, K. pneumoniae and S. aureus*. Further studies are needed to confirm these findings outside Europe and investigate the processes that could causally link income inequality and antimicrobial resistance.

## Introduction

Antimicrobial therapy has contributed greatly to improvements in human health care through the treatment of infectious diseases. Unfortunately, an increase in antimicrobially resistant microorganisms threatens to limit the effectiveness of antimicrobial therapy, and therefore the benefits derived from these drugs [1]. For example, the European Centre for Disease Control recently estimated that 25,000 deaths per year within Europe are directly related to antibiotic resistance [2].

Rates of antimicrobial resistance vary significantly between countries. These variations have been well documented and have been principally related to antimicrobial consumption, i.e. the more antibiotics a country consumes the more resistance is likely to result [3]. However there is limited research to fully explain why resistance rates vary between countries. One area that has yet to be researched is the impact of a nation’s economic policy on antimicrobial consumption and the development of antimicrobial resistance [4]. A nation’s economic policy determines many outcomes within the societies they impact on, including that country’s income inequality. Income inequality is the difference in incomes between those with the highest and lowest incomes in society. Income inequality at the national level has been proposed as being the underlying cause of numerous undesired outcomes in developed countries, e.g. homicide rates, and particularly undesired health care outcomes [5]. Wilkinson and Pickett have shown that higher rates of diabetes and mental illness (including drug and alcohol addiction) are associated with higher levels of income inequality at the national level [5], within developed countries (data from developing countries are unavailable to make similar comparisons). This raises the question of whether income inequality is associated with infection-related health care outcomes, such as antimicrobial resistance. A causative mechanism might be, for example, that with increased levels of disease, e.g. diabetes, there may be a corresponding increase in infections e.g. diabetic foot infections. This could result in more antimicrobial prescribing, in turn associated with higher rates of antimicrobial resistance [3]. This study therefore investigated the associations between income inequality, antimicrobial resistance and antimicrobial consumption within developed countries, via calculation of correlation coefficients between these variables.

## Methods

Income inequality, antimicrobial resistance and antimicrobial consumption were correlated with each other. The analyses were completed on European countries over an eight year time period, 2003-10.

### Income inequality data

Data were taken from the Standardized World Income Inequality Database (SWIID) [6]. The SWIID provides estimates of income inequality within a country which is suitable for broad cross-national analyses. The higher the SWIID score the greater a country’s income inequality.

### Antimicrobial resistance

Data were taken from the European Antimicrobial Resistance Surveillance Network (EARS-Net) [7]. Countries report on the susceptibility of isolates derived from blood cultures and cerebrospinal fluids only. They report data for *Enterococcus faecalis, Enterococcus faecium, Escherichia coli, Klebsiella pneumoniae, Pseudomonas aeruginosa, Staphylococcus aureus* and *Streptococcus pneumoniae*.

### Antimicrobial consumption

Data were taken from the European Surveillance of Antimicrobial Consumption (ESAC) group [8]. ESAC provide data on the defined daily doses (DDD) of antibiotic consumption per inhabitant per 1000 days (DID). DDD is the assumed average maintenance dose per day for a drug used for its main indication in adults, as defined by the World Health Organization [9]. Total antimicrobial consumption data (all antimicrobial classes combined) from outpatients were extracted for the period 2003-2009; consumption data for 2010 data were unavailable. It was not possible to compare a specific antibiotic’s consumption to its correlated resistance as the ESAC data do not correspond directly with the EARSnet data e.g. ESAC reports on penicillin consumption but not on aminopenicillins consumption specifically [10]. Insufficient hospital consumption data from these periods were available for analysis.

### Country and year selection

Only European countries were included in the study as only these countries had comparable antimicrobial resistance data. Countries were selected from the richest 50 (by Gross National Income per capita at purchasing power parities) in 2002. Countries with a population of less than 3 million were excluded to avoid inclusion of tax havens [5]. The following 15 countries were therefore included in the study: Austria, Belgium, Denmark, Finland, France, Germany, Greece, Ireland, Italy, Netherlands, Norway, Portugal, Spain, Sweden and the United Kingdom. Data were analysed from 2003–2010 as EARSnet data are relatively limited before 2003.

### Statistical analysis

Within each antibiotic group, Spearman’s rank order correlation coefficient (r) was calculated per year between income inequality (SWIID) and percentage resistance. Pooled correlations for each antibiotic group were calculated from individual year correlations within this group via the Schmidt-Hunter method [11], as well as their confidence intervals (CIs) and an associated p-value. The Schmidt-Hunter method under-estimates the correlation when it is greater than 0.5 and therefore provides a conservative estimate. Where *r* values are > 0.8, they are classed as strong, > 0.5 moderate, > 0.2 weak and 0 as no association [12]. The CI is given as a range of values which is likely to contain the probable correlation in the general population; a CI not containing 0 indicates sufficient evidence that there is a real correlation in the data. The p-value states how much weight of evidence there is for the observed data, when the true correlation in the population is 0; the smaller the p-value, the more likely a real correlation. These methods were repeated to provide pooled correlations between income inequality and antimicrobial consumption and antimicrobial consumption and antimicrobial resistance. EARS-Net data was incomplete, not every country reported resistance data for all pathogen-resistance combinations every year of the study. Therefore pooled correlations of antimicrobial consumption and antimicrobial resistance were calculated for the subset in which these data were available.

Scatter plots of resistance vs. income inequality by country and year were created for a selection of the antimicrobial resistance and income inequality correlations, with lines of best fit given, per year. Data were initially compiled using Microsoft Excel and were analysed using Stata/IC version 11 and StatsDirect version 3. Graphs were created in Stata/IC version 11.

## Results

Correlations between income inequality, antimicrobial resistance and antimicrobial consumption within European countries are given below, and in Tables 1 and 2.

**Table 1:** Antimicrobial resistance associated with income inequality.

Bacterial species	Antibiotic group	No. years with data available	No. of countries for annual correlations (minimum-maximum)	Pooled correlation	95% Confidence Interval	p-value
*E. faecalis*	Aminopenicillins	8	13-15	0.54	0.49 to 0.60	<0.0001
	High level gentamicin	8	13-15	0.62	0.55 to 0.69	<0.0001
	Vancomycin	8	13-15	0.73	0.68 to 0.79	<0.0001
*E. faecium*	Aminopenicillins	8	13-15	0.26	0.06 to 0.44	0.012
	High level gentamicin	8	13-15	0.38	0.17 to 0.58	<0.0001
	Vancomycin	8	13-15	0.72	0.64 to 0.80	<0.0001
*E. coli*	Aminoglycosides	8	14-15	0.84	0.80 to 0.88	<0.0001
	Aminopenicillins	8	14-15	0.73	0.70 to 0.77	<0.0001
	Carbapenems	8	9-14	0.34	0.18 to 0.49	<0.0001
	Third generation cephalosporins	8	14-15	0.70	0.68 to 0.73	<0.0001
	Fluoroquinolones	8	14-15	0.71	0.60 to 0.82	<0.0001
*K. pneumoniae*	Aminoglycosides	6	13-15	0.50	0.42 to 0.57	<0.0001
	Carbapenems	6	11-15	0.33	0.29 to 0.37	<0.0001
	Third generation cephalosporins	6	12-15	0.57	0.54 to 0.60	<0.0001
	Fluoroquinolones	6	13-15	0.50	0.42 to 0.57	<0.0001
*P. aeruginosa*	Amikacin	6	9-11	0.21	0.15 to 0.26	<0.0001
	Aminoglycosides	6	11-15	0.51	0.47 to 0.56	<0.0001
	Carbapenems	6	11-15	0.50	0.45 to 0.55	<0.0001
	Ceftazidime	6	11-15	0.51	0.47 to 0.55	<0.0001
	Fluoroquinolones	6	11-14	0.53	0.48 to 0.58	<0.0001
	Piperacillin-tazobactam	6	11-15	0.46	0.37 to 0.55	<0.0001
*S. aureus*	Methicillin	8	15	0.86	0.83 to 0.89	<0.0001
	Rifampicin	8	10-13	0.56	0.52 to 0.60	<0.0001
*S. pneumoniae*	Penicillin	8	12-14	0.34	0.25 to 0.43	<0.0001
	Macrolides	8	12-14	0.28	0.08 to 0.47	<0.0001

**Table 2:** Antimicrobial resistance associated with antimicrobial consumption.

Bacterial species	Antibiotic group	No. years with data available	No. of countries for annual correlations (minimum-maximum)	Pooled correlation	95% Confidence Interval	p-value
*E. faecalis*	Aminopenicillins	7	13-15	0.32	0.24 to 0.39	<0.0001
	High level gentamicin	7	13-15	0.30	0.20 to 0.39	<0.0001
	Vancomycin	7	13-15	0.66	0.62 to 0.70	<0.0001
*E. faecium*	Aminopenicillins	7	13-15	-0.06	-0.21 to 0.10	0.47
	High level gentamicin	7	13-15	0.13	0.03 to 0.22	0.013
	Vancomycin	7	13-15	0.51	0.45 to 0.57	<0.0001
*E. coli*	Aminoglycosides	7	14-15	0.49	0.38 to 0.59	<0.0001
	Aminopenicillins	7	14-15	0.27	0.22 to 0.33	<0.0001
	Carbapenems	7	9-14	0.30	0.06 to 0.55	0.015
	Third generation cephalosporins	7	14-15	0.43	0.32 to 0.53	<0.0001
	Fluoroquinolones	7	14-15	0.29	0.19 to 0.38	<0.0001
*K. pneumoniae*	Aminoglycosides	5	13-15	0.84	0.75 to 0.93	<0.0001
	Carbapenems	5	11-15	0.77	0.72 to 0.81	<0.0001
	Third generation cephalosporins	5	12-15	0.85	0.80 to 0.90	<0.0001
	Fluoroquinolones	5	13-15	0.83	0.73 to 0.92	<0.0001
*P. aeruginosa*	Amikacin	5	9-11	0.83	0.73 to 0.92	<0.0001
	Aminoglycosides	5	11-15	0.89	0.87 to 0.92	<0.0001
	Carbapenems	5	11-15	0.83	0.76 to 0.90	<0.0001
	Ceftazidime	5	11-15	0.82	0.79 to 0.85	<0.0001
	Fluoroquinolones	5	11-14	0.84	0.83 to 0.86	<0.0001
	Piperacillin-tazobactam	5	11-15	0.84	0.78 to 0.90	<0.0001
*S. aureus*	Methicillin	7	15	0.67	0.65 to 0.69	<0.0001
	Rifampicin	7	10-13	0.63	0.56 to 0.69	<0.0001
*S. pneumoniae*	Macrolides	7	12-14	0.86	0.83 to 0.90	<0.0001
	Penicillin	7	12-14	0.45	0.37 to 0.52	<0.0001

### Income inequality and Enterococcal resistance

**Figure 1:**
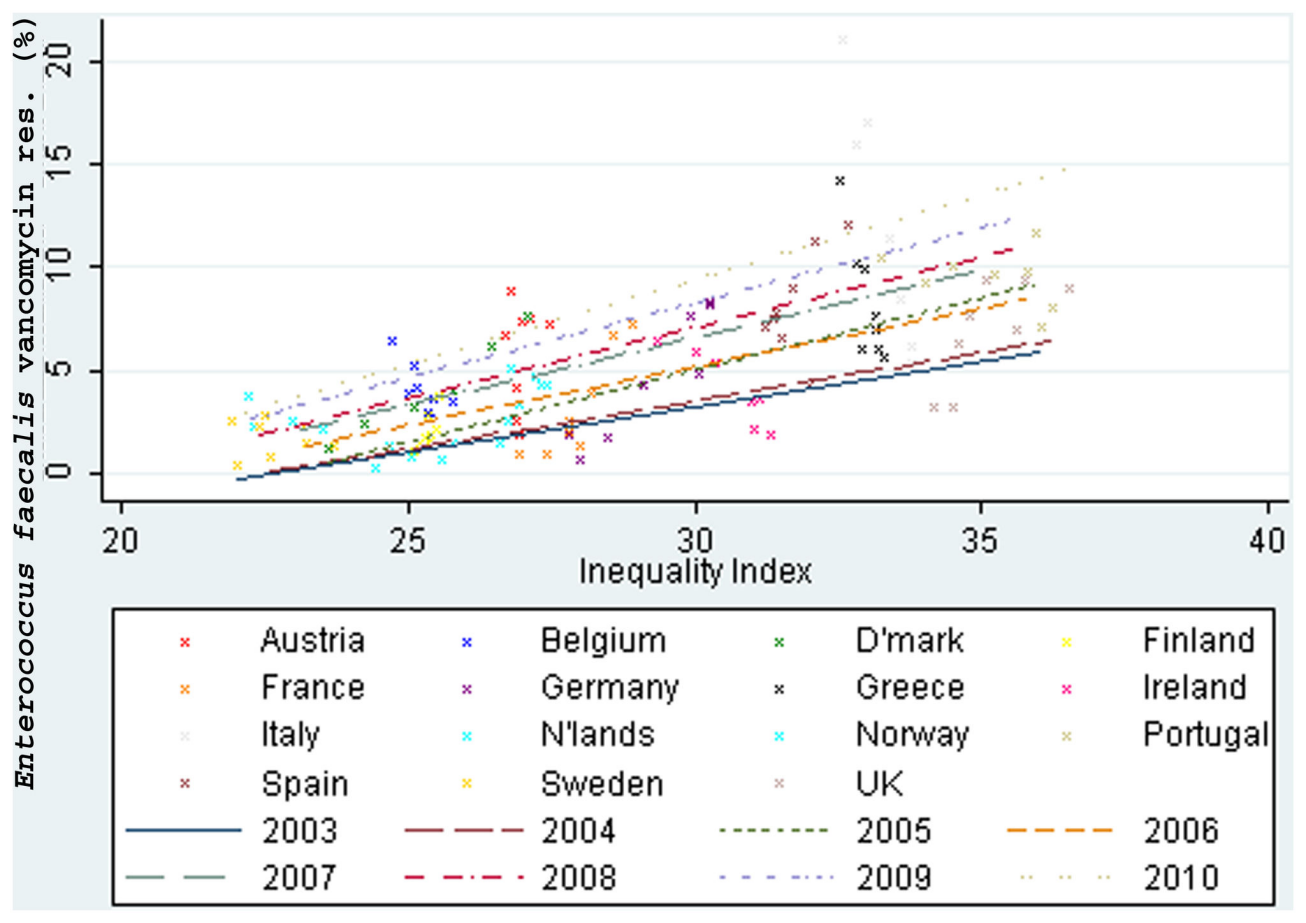
Income inequality correlated with vancomycin resistance in *Enterococcus faecalis* (2003-10).

*E. faecalis* resistance to aminopenicillins was moderately associated with income inequality, *r*=0.54, 95% confidence interval (CI) 0.49 to 0.6. It was also moderately associated with vancomycin resistance, 0.73, 0.68 to 0.79 (Figure 1) and high level gentamicin resistance, 0.62, 0.55 to 0.69. *E. faecium* vancomycin resistance was moderately associated with income inequality, 0.72, 0.64 to 0.80. *E. faecium* resistance to high level gentamicin and aminopenicillins was weakly associated with income inequality, 0.38, 0.17 to 0.58 and 0.26, 0.06 to 0.44 respectively.

### Income inequality and *E. coli* resistance

**Figure 2:**
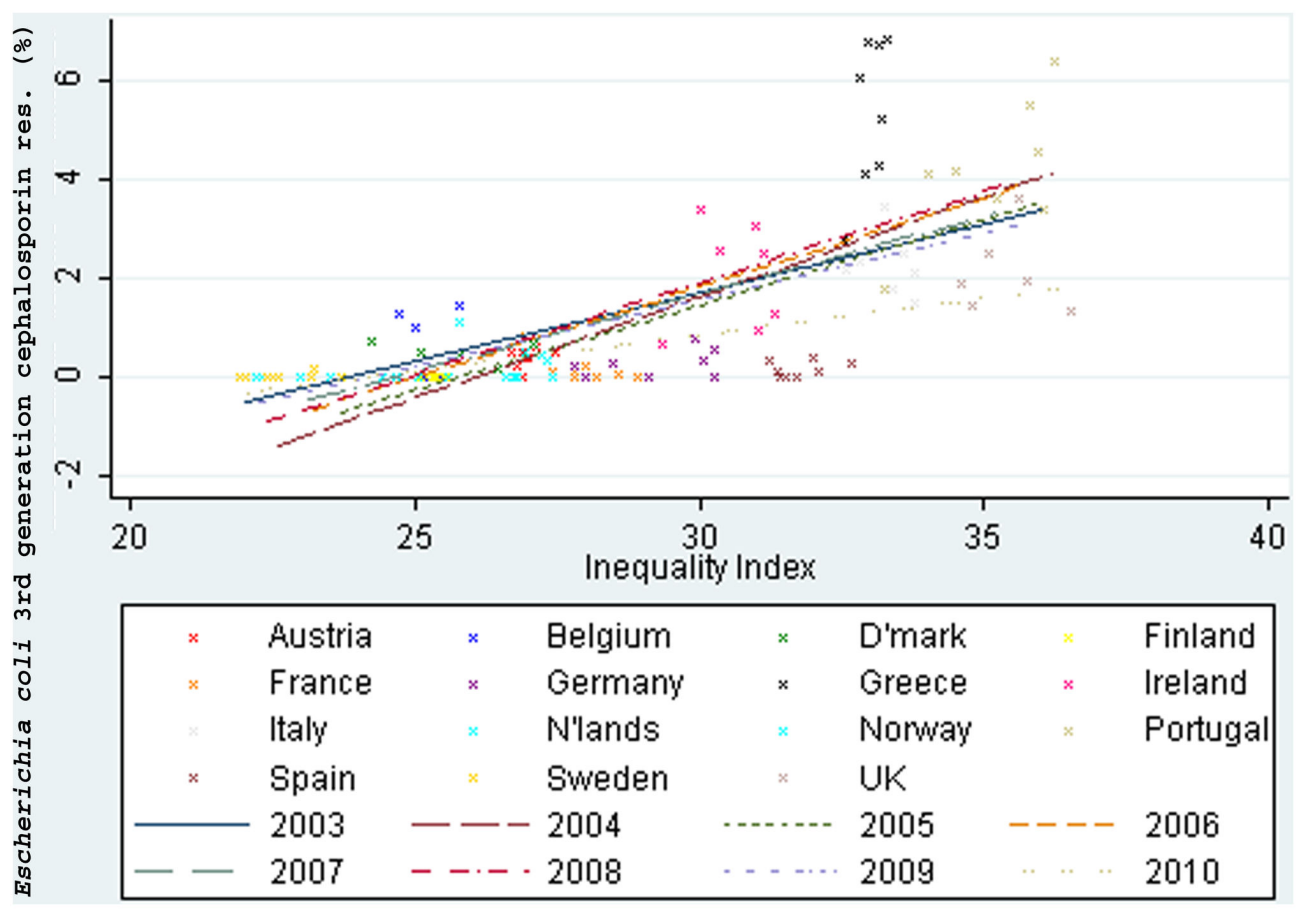
Income inequality correlated with third generation cephalosporin resistance in *Escherichia coli* (2003-10).

*E. coli* resistance to aminoglycosides was strongly associated with income inequality, *r*=0.84, 95% CI: 0.80 to 0.88. Resistance to aminopenicillins, fluoroquinolones and 3^rd^ generation cephalosporins (Figure 2) was moderately associated with income inequality, 0.73, 0.70 to 0.77; 0.71, 0.6 to 0.82 and 0.7, 0.68 to 0.73 respectively. Resistance to carbapenems was weakly associated with income inequality, 0.34, 0.18 to 0.49.

### Income inequality and *K. pneumoniae* resistance

**Figure 3:**
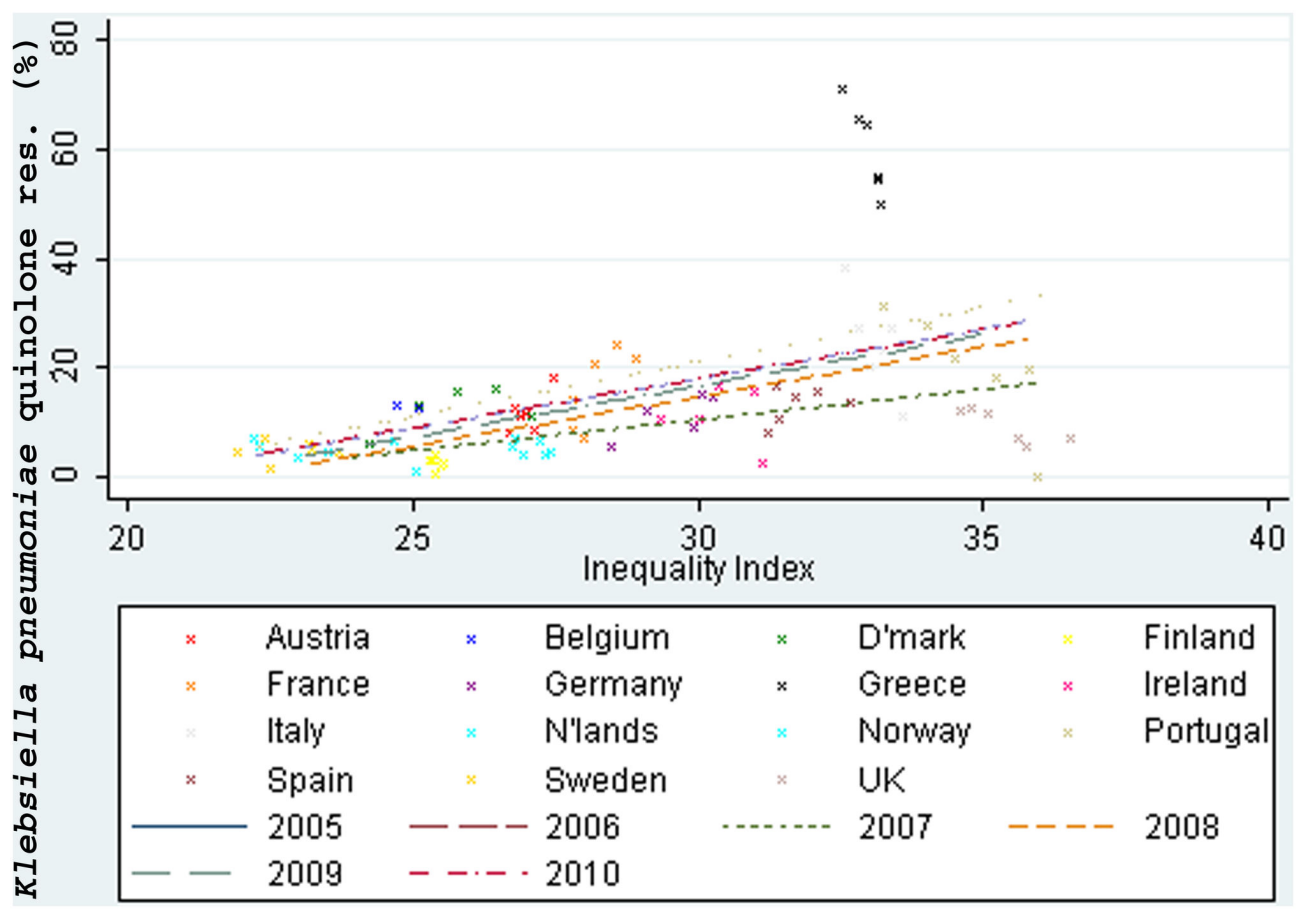
Income inequality correlated with quinolone resistance in *Klebsiella pneumoniae* (2005-10).

*K. pneumoniae* resistance to aminoglycosides, third generation cephalosporins and fluoroquinolones (Figure 3) was moderately associated with income inequality, *r*=0.50, 95% CI: 0.42 to 0.57; 0.57, 0.54 to 0.60 and 0.50, 0.42 to 0.57 respectively. Carbapenem resistance was weakly associated with income inequality, 0.33, 0.29 to 0.37.

### Income inequality and *P. aeruginosa* resistance

*P. aeruginosa* aminoglycoside, carbapenem, ceftazidime and fluoroquinolone resistance was moderately associated with income inequality, *r*=0.51, 95% CI: 0.47 to 0.56; 0.50, 0.45 to 0.55; 0.51, 0.47 to 0.55 and 0.53, 0.48 to 0.58 respectively. Piperacillin-tazobactam and amikacin resistance were weakly associated with income inequality, 0.46, 0.37 to 0.55 and 0.21, 0.15 to 0.26 respectively.

### Income inequality and *S. aureus* resistance data

**Figure 4:**
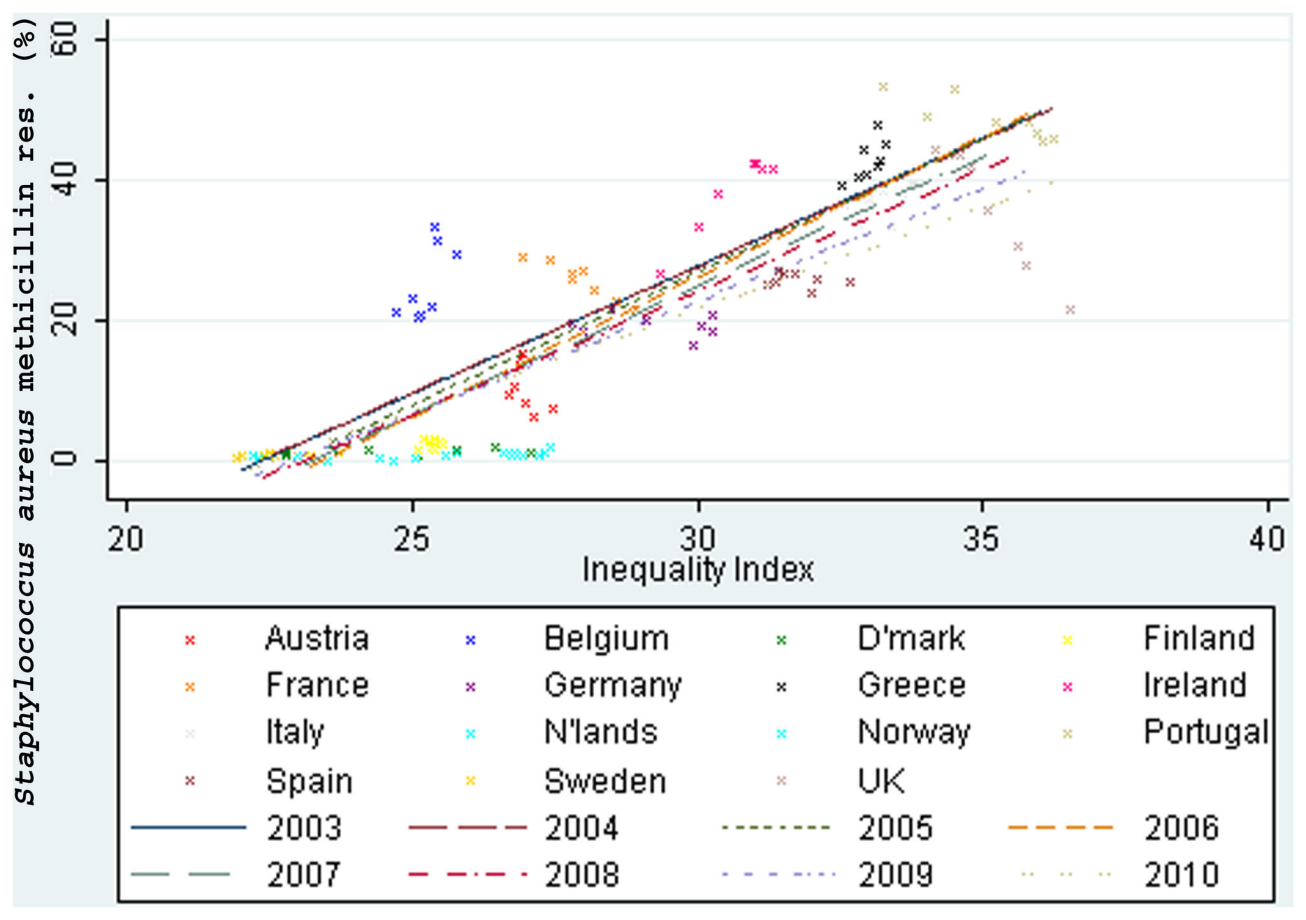
Income inequality correlated with methicillin resistance in *Staphylococcus aureus* (2003-10).

*S. aureus* methicillin resistance and income inequality were strongly associated, *r*=0.86, 95% CI: 0.83 to 0.89 (Figure 4). *S. aureus* rifampicin resistance was moderately associated with income inequality, 0.56, 0.52 to 0.60.

### Income inequality and *S.* *pneuomoniae* :

*S. pneumoniae* resistance to penicillin and macrolides was weakly associated with income inequality, *r*=0.34, 95% CI: 0.25 to 0.43 and 0.28, 0.08 to 0.47.

### Income inequality and antimicrobial consumption

Antimicrobial consumption in outpatients measured by Defined Daily Doses per 1000 inhabitants per day (DID) was weakly associated with income inequality, *r*=0.44, 95% CI: 0.41 to 0.48.

### Antimicrobial consumption and antimicrobial resistance

Total antimicrobial consumption in outpatients, measured by Defined Daily Doses per 1000 inhabitants per day (DID), was strongly associated with resistance in the following species-resistance pairings: *K. pneumoniae*-aminoglycosides, *r*=0.84, CI: 0.75 to 0.93, *K. pneumoniae*-third generation cephalosporins, 0.85, 0.80 to 0.90, *K. pneumoniae*-fluoroquinolones, 0.83, 0.73 to 0.92, *P. aeruginosa*-amikacin, 0.83,0.73 to 0.92, *P. aeruginosa*-aminoglycosides, 0.89, 0.87 to 0.92, *P. aeruginosa*-carbapenems, 0.83, 0.76 to 0.90, *P. aeruginosa*-ceftazidime, 0.82, 0.79 to 0.85, *P. aeruginosa*-piperacillin-tazobactam, 0.84, 0.78 to 0.90 and *S. pneumoniae*-macrolides, 0.86, 0.83 to 0.90. All other bacterial-resistance pairings analysed were weakly or moderately associated with antimicrobial consumption except for *E. faecium*-aminopenicillins which was not associated with antimicrobial consumption, -0.06, -0.21 to 0.10 (Table 2).

## Discussion

This study showed income inequality to be positively correlated with antimicrobial resistance in all seven species analysed: *E. faecalis*, *E. faecium*, *E. coli*, *K. pneumoniae*, *P. aeruginosa*, *S.* aureus* and S. pneumoniae*. In addition, inequality was correlated with antimicrobial consumption, and antimicrobial consumption was correlated with antimicrobial resistance.

Strengths of the data analysed in this study include resistance data, antimicrobial consumption data and inequality data being collected by single sources: EARS-Net, ESAC and SWIID, and there being no selection bias in this study as all EARS-net data were analysed. Limitations of the data are: the data is only from developed countries within Europe and data are estimates only. The antimicrobial consumption data used consisted of total out-patient antibiotic data (all classes combined), and this study looks at in-hospital bacterial resistance to specific antibacterials. It has though been shown that out-patient antibiotic consumption is correlated with in-patient antibiotic consumption [13] and antimicrobial consumption for a specific antimicrobial has been related to resistance to that antimicrobial in previous studies [3]. The correlations between income inequality and antimicrobial resistance, seen across all pathogens studied (n=7) and all antimicrobials (n= 14), are not independent of each other in all cases. This is principally an issue in *E. coli* and *K. pneumoniae* due to plasmid-mediated resistance, with plasmids carrying resistance to more than one antimicrobial class [14].

Only correlations are reported in this study and therefore no causality can be proven. Investigating if causality is present between inequality and resistance is not possible given the data only allows us to calculate correlations, though these correlations reported are consistent with our hypothesis. It is important to consider if biologically plausible causal mechanisms support the correlations. Antimicrobial resistance rates can be increased in two ways. Firstly, exposure to antimicrobials can create resistance de novo and select out resistant strains. Secondly, transmission of antimicrobially resistant bacteria can occur. The associations between income inequality and antimicrobial consumption, and between antimicrobial consumption and antimicrobial resistance, support the former mechanism. In the case of *E. faecium*-aminopenicillin resistance, where there is no correlation between consumption and resistance, as would be expected, there was still a correlation, albeit weak, between income inequality and antimicrobial resistance. This would support the latter mechanism, i.e. that income inequality is involved in resistance through transmission of resistant bacteria, although no mediating factor analogous to antimicrobial consumption has been identified.

To support causality between inequality and consumption, mediating factors are needed, of which there are many possibilities. For example, increased income inequality is associated with higher Body Mass Index (BMI) [5]. Higher BMIs are associated with comorbidities such as diabetes, resulting in higher rates of skin infections, caused by *S. aureus*, and urinary tract infections, caused by *E. coli* [15,16]. Co-morbidities, e.g. obesity, could theoretically result in more severe infections. However, there is evidence that antimicrobial consumption varies independently of infection severity [17]. Given this, there could be factors not related to severity that income inequality impacts upon which affect antibiotic consumption. For example, increased income inequality is associated with lower levels of education, and lower educational levels have been associated with higher rates of antimicrobial consumption [5,18]. A defined dose of an antibiotic may also result in varying risks of developing antibiotic resistance in different countries. For example, patients with higher BMIs have lower antibiotic concentrations in the body. These lower concentrations have been implicated in the development of antibiotic resistance, sub-therapeutic drug concentrations failing to prevent the emergence of resistant bacteria [19,20]. Income inequality is also correlated with factors likely to result in an increased transmission of drug resistance e.g., imprisonment is associated with community associated-MRSA and Multi-Drug Resistant *Mycobacterium tuberculosis* [5,21,22].

Assuming there is causality between income inequality and antimicrobial resistance, the mediating factor which seems likely to be most important is considered to be antimicrobial consumption. A simple triangular model between inequality-consumption-resistance may not though reflect reality. As already demonstrated, *E. faecium* resistance is correlated with inequality even though consumption and resistance and not correlated for this antibiotic. Consideration of the relative strengths of the correlations also challenges this simple model. The correlations between income inequality and antimicrobial resistance are strongest for *S. aureus* and *E. coli*. But the strongest relationships between antimicrobial consumption and antimicrobial resistance are strongest in *K. pneumoniae* and *P. aeruginosa*. Inequality is likely to act differently on bacteria depending if they are more commonly community or hospital pathogens, for example, *S. aureus* and *E. coli* are considered common community pathogens when compared to *K. pneumoniae* and *P. aeruginosa*. Also, consumption affects different bacteria differently. For example, *Klebsiella* *spp* more easily develop cephalosporin resistance compared to *E. coli* due to their chromosomal carriage of *B*-lactamases [23].

Weak correlations between inequality and resistance were identified for some pathogen-antimicrobial pairings. The weak association between aminopenicillin antimicrobial resistance in *E. faecium* with income inequality could be expected, as *E. faecium* is commonly resistant to aminopenicillins [24], whereas all other species are typically susceptible to the antibiotics they are tested against. *S. pneumoniae* resistance has been affected by the introduction of *S. pneumoniae* vaccination, causing a replacement of antimicrobial resistance serotypes of *S. pneumoniae* [25]. This vaccine effect may have attenuated links between *S. pneumoniae* resistance and income inequality, though no data support this suggestion. For carbapenems there may be temporal effects present, as these are the most recently introduced class of antibiotics studied.

The associations described here do not tell us how increased levels of resistance are distributed within populations as they are based on population statistics. That is, to suggest these data allow us to say if resistance is more common in the rich or poor is not possible. To do so would be to make a statistical inference error known as the “ecological fallacy”. The ecological fallacy occurs when statistical data collected about a group is incorrectly used to make inferences about the nature of individuals e.g. rich or poor. There is some evidence available on who may be affected within the population. It may be that the relatively poor within a society suffer most from increased antimicrobial resistance as their increased rates of disease e.g. diabetes, make them prone to more infections [26]. However, within less equal societies the rich suffer increased rates of disease compared to the rich within more equal societies [27]. Therefore, as income inequality increases, the rich may have relatively high rates of antibiotic resistance. This association has in fact been shown in a number of studies. In the USA, households with higher median incomes had higher rates of infection with penicillin non-susceptible pneumococci (PNSP) [28]. In Sweden, PNSP have been positively correlated with antibiotic prescribing, which has been positively correlated with per capita income [29]. In the USA study most disease was seen in the relatively poor, but resistance was higher in the relatively rich. It is likely that access to healthcare as well as the presence of an infection is important in the risk of antimicrobial resistance; a study in Ireland showed patients treated privately were prescribed antibiotics more often than those treated by the public health system [30].

The associations between income inequality and antimicrobial resistance should be considered in the context of other factors which impact on antimicrobial resistance, e.g., culture and practices of prescribers and the public, infection control practices, intensity of migration and practices of antibiotic use in the agricultural sector [31–33]. These factors may be independently associated with antimicrobial resistance and potentially income inequality; therefore possible confounding by income inequality should always be taken into account in further antibiotic resistance research. It is likely there is a contribution from numerous factors to a country’s overall level of antimicrobial resistance and that income inequality, if causality exists, is one of these factors.

Before undertaking this study, correlations between income inequality and antimicrobial resistance had not been quantified. This evidence for a positive association of income inequality and antimicrobial resistance provides the best information available at present with which to consider if increasing income inequality in developed countries does increase antimicrobial resistance; this should be considered by those researching why some countries are better at preventing and controlling the emergence of antimicrobial resistance. To investigate these associations further requires more data and these may come from two areas. Firstly, it may be possible to encourage agencies outside Europe to collect data comparable to that of the EARS-net, e.g., Centers for Disease Control (U.S.A.) could complete state-based surveillance. Secondly, it may also be possible to monitor how the correlation between income inequality and antimicrobial resistance changes temporally within a country.

It is important for a country to know what to consider a normal level of resistance, and using income inequality correlations may allow this to be achieved. Knowing expected resistance rates is important as organisational reforms may be carried out based on crude rates, without consideration of expected rates. In the UK for example, reforms to the National Health Service (NHS) have been introduced, one of the justifications being that the UK had a higher incidence of MRSA than the European average [34]. If resistance and inequality are causally related then the UK had MRSA rates consistent with its level of income inequality and it would be incorrect to cite past UK MRSA rates in support of reforms to the UKs NHS [34].

In summary, income inequality in developed European countries is associated with antimicrobial resistance. These associations are consistent with a belief that modifying income inequality within a nation may be an effective public health intervention to reduce antimicrobial resistance rates.
